# A Clinical Trial of the Effects of a Dietary Pattern on Health Metrics and Fecal Metabolites in Volunteers With Risk of Cardiovascular Disease

**DOI:** 10.3389/fnut.2022.853365

**Published:** 2022-05-10

**Authors:** Kunchen Han, Jinke Ma, Junxia Dou, Dan Hao, Wenjun Zhu, Xiaohan Yu, Wenxuan Zheng, Yao Song, Fengcui Shi, Quanyang Li

**Affiliations:** ^1^School of Light Industry and Food Engineering, Guangxi University, Nanning, China; ^2^Department of Biotechnology Engineering, Taishan Polytechnic, Taian, China; ^3^Department of Pharmacology and Nutritional Science, University of Kentucky, Lexington, KY, United States

**Keywords:** dietary patterns, biochemical parameters, fecal metabolomics, cardiovascular diseases, prebiotic effects

## Abstract

The phenomenon of longevity in Guangxi of China proved to be closely relevant to its specific dietary habits, but the exact effects of this diet on health remain to be explored. In this work, fourteen screened volunteers with cardiovascular disease (CVD) risk followed a novel dietary pattern derived from centenarians of Guangxi, China for 2 weeks, then the effects of diet on human health were explored by measuring the health metrics and fecal metabolites. The results showed that the short-term dietary intervention significantly decreased the body weight, body mass index (BMI), total cholesterol (TC), low-density lipoprotein cholesterol (LDL-c), mean systolic blood pressure (SBP), and diastolic blood pressure (DBP) levels, while it significantly increased high-density lipoprotein cholesterol (HDL-c) levels. Orthogonal partial least squares discriminant analysis (OPLS-DA) indicated a distinct separation in the fecal metabolic profiles of volunteers before and after the intervention. Nine of these metabolites showed significant differences, including two metabolites increased (butyrate and citrulline), seven metabolites decreased (threonine, choline, glycine, aspartate, alanine, N-acetylglutamic acid and lysine). Pathway and enrichment analysis showed that the reduction in CVD risk by dietary intervention mainly affected five pathways, which include arginine biosynthesis; aminoacyl-tRNA biosynthesis; glycine, serine and threonine metabolism; alanine, aspartate and glutamate metabolism; and valine, leucine and isoleucine biosynthesis. Herein, the Guangxi longevity dietary pattern can provide a feasible healthy diet strategy for reducing the CVD risk and human beings.

**Clinical Trial Registration:**

[http://www.chictr.org.cn], identifier [ChiCTR220 0058216].

## Graphical Abstract

Schematic diagram of the study design (2 weeks dietary intervention, single arm intervention).

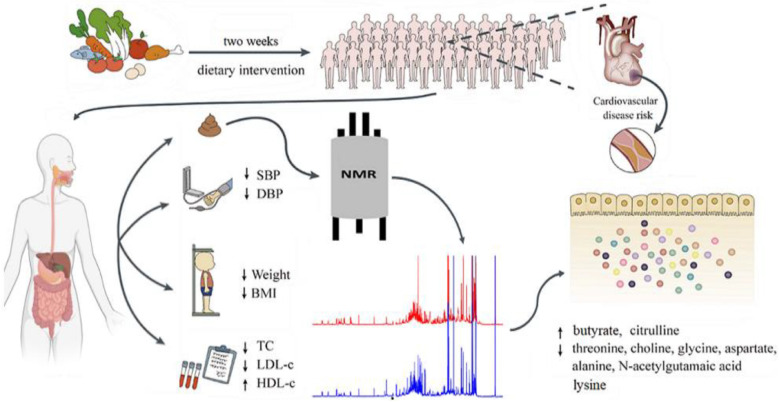

## Introduction

Cardiovascular disease (CVD) is the leading cause of death worldwide. A report has shown that 18.6 million people died of CVD-related diseases in 2019 (1). A healthy diet has been proven to be effective in reducing the risk of CVD and helping people live healthier lives (2, 3). Studies have shown that a high dietary fiber intake is significantly associated with a reduction in the risk of coronary heart disease, type 2 diabetes and CVD mortality, etc. (4). Higher intakes of fruits, vegetables, and beans are inversely associated with the risk of CVD (5). Interventions regarding overall dietary patterns rather than a single food or nutrient are a more effective way of preventing or reducing CVD risk, as a healthy dietary pattern focuses more on the rational selection and matching of foods so that different nutrients may create synergistic effects in the body, which can help the body to better digest and absorb food. Moreover, the report found a negative correlation between continuous improvement in overall diet quality and mortality (6, 7). Therefore, studying dietary patterns is a practicable entry point to reduce CVD risk and promote healthy aging of the organism (8, 9). Considering that major crops and dietary habits vary markedly in different countries and regions, the associations between different dietary patterns and CVD risk factors are also different. At present, relevant diversity studies are very limited. Therefore, a focus on healthy diets in specific regions to help populations maintain body health status and reduce CVD risk is urgently needed.

The phenomenon of longevity in Guangxi, China, is remarkable, with 31 “Longevity towns of China”, accounting for more than 35% of the total number in China (10). The number of longevous populations in this area has been increasing in recent years. Studies have found that the local centenarians are healthy, with higher high-density lipoprotein cholesterol (HDL-c) levels, lower triglycerides (TG) levels and dyslipidemia prevalence, and have a lower risk of CVD (11). Our team explored and traced the factors that contributed to its longevity from the perspective of diet over many years. After studies on dietary and metabolite characteristics, gut microbiota and other aspects of the longevity population cohort in the longevity population densely-occupied area of Guangxi, found that the diet of the elderly in this area was rich in high dietary fiber polysaccharides, dominated by porridge, coarse-grained, dark vegetables and livestock meat, which is characterized by its high dietary fiber, low energy, low fat, low protein, and low cholesterol content (12, 13). From this, we constructed the Guangxi longevity dietary pattern which was validated to have good effects on reducing inflammation and resisting aging on D-galactose-induced aging mice and naturally aging mice (14, 15). Based on this evidence, we tried to explore the positive effects of Guangxi longevity dietary pattern on human health. We recruited volunteers to conduct pre-experiments and found that it played a good role in improving volunteers’ body weight, especially blood lipid, and had a good potential to improve the body’s CVD risk. Based on these preliminary data, this study was conducted to explore the exact effect on reducing the risk of CVD in humans by recruiting volunteers with high compliance to this dietary pattern intervention.

Metabolomic profiling has become an important tool, used to understand the interactions between metabolites and unveil the mechanism of organism health at the biochemical level. Feces, as the final product of cellular and microbial metabolism in the intestinal tract of the body, is one of the best biomaterials to reflect the information of physiological changes in the gut (16). Profiling fecal metabolomics has become an important way of exploring the association between diet, human metabolism, and gut microbiota composition when maintaining the healthy state of the organism, and helps to clarify the physiological and biochemical mechanisms of dietary and metabolic changes in the body (17). Nuclear magnetic resonance (NMR), an important analysis method in metabolomics detection, has the characteristics of high reproducibility and being non-destructive to samples (18). Therefore, detecting the changes in fecal metabolites caused by dietary intervention through ^1^H NMR fecal metabolomics was valuable, providing a possible way for the relationship between dietary intervention and the beneficial effects on cardiovascular health.

The objectives of this study were to examine the impact of the constructed dietary pattern on anthropometric measured, biochemical parameters and fecal metabolites in volunteers with a high risk of CVD, and evaluate its role as a means of reducing CVD risk and promoting cardiometabolic health.

## Materials and Methods

### Study Design

This was a 2-weeks single-arm intervention study, whereby participants acted as their own control. Volunteers were required to strictly follow the designed dietary pattern for 2 weeks. The study was approved by the Ethics Committee of Guangxi University (Approval No.GXU-2020-136). At the beginning of the study, volunteers were informed of the contents and potential risks involved in the experiments and signed a “volunteer informed consent”. Meanwhile, this study was retrospectively registered at the Chinese Clinical Trial Registry (http://www.chictr.org.cn) (registration number: ChiCTR2200058216).

#### Power Calculation

This study considered systolic blood pressure (SBP) as the primary outcome measure (19). The power calculation was carried out using R Project. Based on SBP levels measured in previous studies of similar dietary interventions (20), assuming mean SBP difference of 6 mmHg and standard deviation of 7 mmHg, a level of significance of 0.05, we calculated that 13 subjects were required for a statistical power of 80%.

#### Participant Recruitment

**FIGURE 1:**
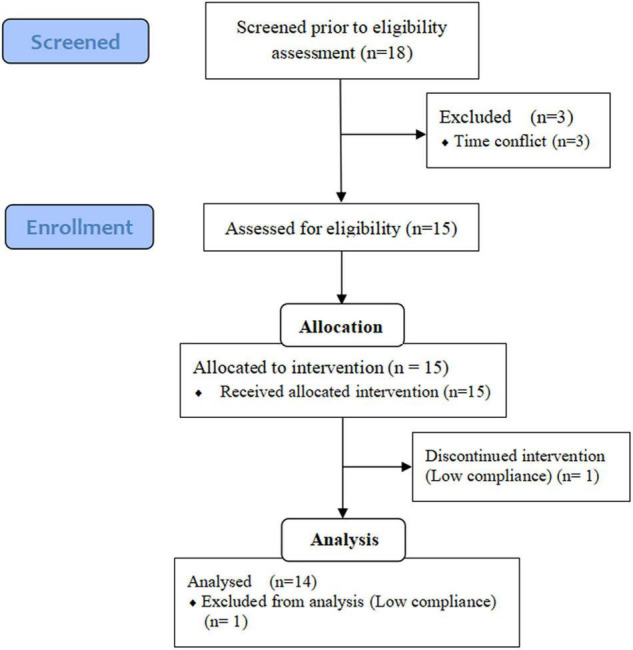
Flow diagram of the study selection process. Adapted from (21).

Volunteers were recruited from October 8, 2020, to December 1, 2020, through leaflets, email, and personal communication in Nanning, Guangxi, China. The participants were screened based on the following criteria: not taking medications that chronically affect the immune system; not taking antibiotics during the intervention; no supplementation of prebiotics, probiotics or vitamins for 2 weeks before the experiment; at least one CVD risk factor, including: (a) overweight or obesity (BMI > 25 kg/m^2^); (b) TG > 1.88 mmol/L; (c) total cholesterol (TC) > 5.7 mmol/L; (d) HDL-c < 0.9 mmol/L; (e) low-density lipoprotein cholesterol (LDL-c) > 4.11 mmol/L; (f) fasting blood glucose (FBG) > 6.1 mmol/L; (g) blood pressure > 140/90 mmHg. The flow diagram of study selection process was showed in Figure 1. Eighteen volunteers were eligible for this study. Three volunteers excluded because of emergencies in individual homes, and one volunteer was excluded because of poor compliance. The data were finally collected from 14 participants (age from 50 to 75): 7 males, 7 females. Questionnaires were used to quantify their physical activity (22).

#### Condensation of Nutrients for Guangxi’s Longevity Dietary Pattern

According to the results of the team’s earlier survey on the diet of centenarians in the Guangxi longevity area, based on the characteristics of high dietary fiber, low energy, low fat and low protein (12, 13), combined with the “Chinese Dietary Guidelines summary (2016),” “Chinese Dietary Guideline of the Elderly (2010),” and “the Report on Dietary Nutrition and Health status of Guangxi Residents,” the recommended amount of nutrients and the nutritional content of corresponding foods, with the investigation on the local daily diet, the important nutrients and characteristic foods required for longevity diets were listed, we have constructed the Guangxi longevity dietary pattern in terms of energy, fat, protein, carbohydrate and dietary fiber. Among them: energy intake was calculated based on the recommended amounts of dietary guidelines, combined with the results of the team’s previous animal experiments (23). The dietary fiber intake was calculated based on the optimal dose in mice experiments, combined with the results of dietary surveys on centenarians (12, 15). The intakes of fat, protein, carbohydrates were all designed based on the dietary characteristics of centenarians (12), and optimized in combination with recommended amounts from dietary guidelines, accounting for 15, 25, and 60% of energy intake, respectively. Diet composition was as follows: 1710 ± 38 total Kcal/day, 15% ± 1% protein, 25% ± 2% fat, and 60% ± 2% carbohydrate, including 32.91 ± 0.73 g dietary fiber intake, based on volunteers aged 60–70 weigh 60 kg with a BMI = 21.25.

#### Study Food Development

Each volunteer’s nutrient intake was targeted based on information such as gender, weight, and daily work intensity, obtained from previous surveys and measurements. During the design process, BMI = 21.25 (median of 18.5–24.0) was used as the standard to determine their total energy intake based on the energy requirements for different genders, ages, and physical activities recommended in the “Chinese Dietary Guidelines summary (2016)” and “Chinese Dietary Guideline of the Elderly (2010),” combined with the requirements of the Guangxi longevity dietary pattern. Protein, fat, carbohydrate, and dietary fiber intakes were calculated based on energy and according to the intake of each nutrient designed in the Guangxi longevity dietary pattern. For each volunteer, on the basis of the same gender, age, and level of physical activity, the energy intake was calculated by multiplying the coefficient based on the ratio of basal metabolic rate between the volunteer’s BMI and the standard BMI. Meanwhile, for the obese volunteers (BMI > 28), we set an obesity coefficient of 0.95 to control their total energy intake. The menus are based on our nutrient intake, designed according to the volunteers’ situation, combined with the preferred food of the centenarians in the Guangxi longevity area and the characteristic crops grown in this area. The food groups used in the dietary pattern are shown in Supplementary Table 1. The menus were designed to be refined into a 7-day diet, and the recipes were cycled every 7 days. Examples of 1-day menus are given in Supplementary Table 2. The volunteer’s diet was prepared and supplied in a specific place that met the requirements. All food was precisely measured and weighed.

#### Intervention

Volunteers participating in this study were required to keep their eating habits stable for at least 2 weeks before beginning the dietary intervention. During the dietary intervention, volunteers were asked to receive the designed meal for 2 weeks with high compliance. The meal consists of breakfast, lunch and dinner. Except for drinking water, no other food will be consumed. All volunteers were required to maintain a stable level of physical activity during the intervention. Meanwhile, special attention was paid to communicating with volunteers about their compliance and helping them address any specific issues that arose.

### Anthropometric Assessment

The age and sex of the volunteers were recorded before the experiment begins. Volunteers’ height and weight were measured before the experiment, in the middle of the experiment (morning of day 8), and after the experiment (morning of day 15) for the calculation of body mass index [BMI = weight (kg)/height (m^2^)]. The blood pressure of volunteers was measured using a sphygmomanometer. Volunteers were told to avoid intensive exercise 1 h before the measurement and avoid moving and speaking during the measurement.

### Blood Specimen Collection and Measurements of Clinical Parameters

Blood samples from volunteers were collected in anticoagulant tubes using a non-invasive method after an overnight fast at day 0, 8, 15, for the detection of blood indicators. Serum samples were measured for FBG, TC, TG, LDL-c, and HDL-c using an automated biochemistry analyzer (Roche Cobasc702, IN).

### Fecal Untargeted Metabolomics Analysis Based on ^1^H-NMR

#### Sample Preparation

Using a non-invasive method, fecal samples were collected on spontaneous defecation before and after dietary intervention. Feces were immediately put into the icebox and transferred to a −80°C freezer. Fecal metabolites were extracted using the method of Wu et al., with minor modifications (24). In brief, a homogenized 50 mg aliquot was placed into a 2.0 mL safe-lock tube (Eppendorf, Hamburg, Germany), then, 500 μL PBS/D_2_O buffer (0.1 M, pH 7.4) was added, containing 10% D_2_O (v/v) and 0.005% TSP (w/v), vortexed for 15 s, mixed and repeatedly freeze-thawed with liquid nitrogen three times. The mixture was homogenized for 60 s, followed by centrifugation (12,000 *g*, 10 min, 4°C). Supernatants were removed and the extraction procedure was repeated. The supernatants from two extractions were collected for centrifugation (12,000 *g*, 15 min, 4°C). Then, 550 μL of the resulting supernatant was transferred into a 5 mm NMR tube for further analysis.

#### ^1^H-NMR Data Acquisition and Processing

^1^H NMR spectra of the feces were measured by a Bruker Avance 500 MHz NMR spectrometer (Bruker, Germany) at a ^1^H frequency of 500.13 MHz and a temperature of 298 K. Fecal extract samples were recorded using the water-pre-saturated standard one-dimensional NOESYPR1D pulse sequence (recycle delay − 90°− t_1_ − 90°− t_*m*_ − 90°− acquisition) with water suppression to obtain representative total metabolite compositions. The acquisition parameters were as follows: relaxation delay, 2.0 s; mixing time, 0.1 s; number of scans, 64; spectral size, 65,536 points; spectral width, 10,000 Hz. MestReNova (version 14.0, Mestrelab Research, Spain) was used to obtain the NMR spectra. All free induction decays (FIDs) from 1D ^1^H NMR were multiplied by a 0.5 Hz exponential line broadening before Fourier Transformation. After corrections for phase and baseline distortion, ^1^H NMR spectra were referenced to the TSP signal (δ 0.0) and spectral regions 0.00–9.00 ppm were integrated into regions with equal widths of 0.001 ppm. The water signal, dominating the spectrum between 4.70 ppm and 5.10 ppm, was excluded to eliminate the spurious effect of water suppression, and the integration data were normalized for further analysis.

#### Multivariate Data Analysis and Biomarker Identification

SIMCA-P software file (V13.0, Umea, Sweden) was used for multivariate data analysis. An unsupervised pattern recognition analysis method, principal component analysis (PCA) was applied to examine any intrinsic clustering of the data. Furthermore, supervised orthogonal partial least squares discriminant analysis (OPLS-DA) was performed to maximize the separation between metabolites before and after dietary intervention. The validity of the model was assessed with the R^2^Y and Q^2^ values, reflecting the explained variables and the predictability of the model. The validity of the model was further evaluated with rigorous permutation tests (*n* = 200). Feces metabolites with fold change (FC ≥ 1.1 or ≤0.9), paired samples Wilcoxon test *p* < 0.05 and variable importance in projection (VIP) values > 1 were considered potential biomarkers.

### Functional Interpretation and Metabolic Network Construction

Metabolic networks were constructed using the Search Tool for Interactions of Chemicals (STITCH) to illustrate the relationships between biomarkers and relevant targets that modulate the risk of CVD (25). The minimum required interaction score was set to 0.4. The obtained results were downloaded and visualized by the Cytoscape software. On the MetaboAnalyst 5.0 platform, pathway analysis and enrichment analysis based on the Kyoto Encyclopedia of Gene and Genomes (KEGG) database were performed to visualize the fecal metabolite network and confirm the targeted pathways that are involved.

### Statistical Analysis

All the analyses were performed using SPSS version 25.0 (SPSS Inc., United States) and a 5% level of significance. The data were expressed as mean ± SD values. The Mann–Whitney U test was used to the differences in continuous variables between the different sexes. Paired samples Wilcoxon test was used to examine differences in volunteer metabolites before and after dietary intervention and to examine differences in volunteer anthropometric measurements and biochemical indexes from pre-dietary intervention to mid- and post-dietary intervention separately. Pearson correlation analysis was used to examine the relationships between fecal metabolites and clinical parameters before and after the intervention.

## Results

### Anthropometric and Biochemical Parameters of Participants

Table 1 lists anthropometric data and blood biochemical parameters throughout the study period. Compared to the baseline, the body weight, BMI, blood pressure and blood lipid levels of volunteers were significantly improved after the two-week experiment. These indicators decreased significantly in the first week and gradually stabilized in the second week. By the end of the dietary intervention, the volunteer’s body weight decreased by 2.96% (*p* = 0.004), BMI decreased by 2.96% (*p* = 0.004), while SBP and diastolic blood pressure (DBP) decreased by 6.33% (*p* = 0.041) and 3.62% (*p* = 0.044), respectively. TC and LDL-c decreased by 8.84% (*p* = 0.002) and 16.98% (*p* = 0.001), respectively. HDL-c significantly increased by 54.71% (*p* = 0.001). After dietary interventions, the FBG and TG levels also showed a downward trend, but they did not show a statistical difference (*p* > 0.05).

**TABLE 1:** Body composition and biochemical parameters of participants throughout the study^a^.

	Baseline	Day 8	Day 15	*p* Value	
				Baseline vs. Day 8	Baseline vs. Day 15
Weight (kg)	64.72 ± 13.74	62.83 ± 12.92	62.68 ± 12.68	0.002	**0.004**
BMI (kg/m^2^)	25.42 ± 3.75	24.69 ± 3.45	24.63 ± 3.35	**0.002**	**0.004**
TC (mmol/L)	5.36 ± 0.91	5.17 ± 0.79	4.88 ± 0.86	**0.035**	**0.002**
TG (mmol/L)	1.92 ± 0.92	1.64 ± 0.53	1.76 ± 0.55	0.177	0.330
HDL-c (mmol/L)	1.10 ± 0.22	1.84 ± 0.37	1.70 ± 0.44	**0.001**	**0.001**
LDL-c (mmol/L)	3.44 ± 0.63	2.73 ± 0.46	2.84 ± 0.58	**0.001**	**0.001**
FBG (mmol/L)	5.64 ± 1.10	5.39 ± 1.02	5.39 ± 0.83	0.234	0.073
SBP (mmHg)	132.50 ± 20.47	120.43 ± 11.82	122.50 ± 11.22	**0.033**	**0.041**
DBP (mmHg)	79.93 ± 11.84	76.71 ± 11.66	76.64 ± 9.83	0.284	**0.044**

*^a^Data are presented as mean ± SD value. The meaning of the bold value is p < 0.05.*

**FIGURE 2:**
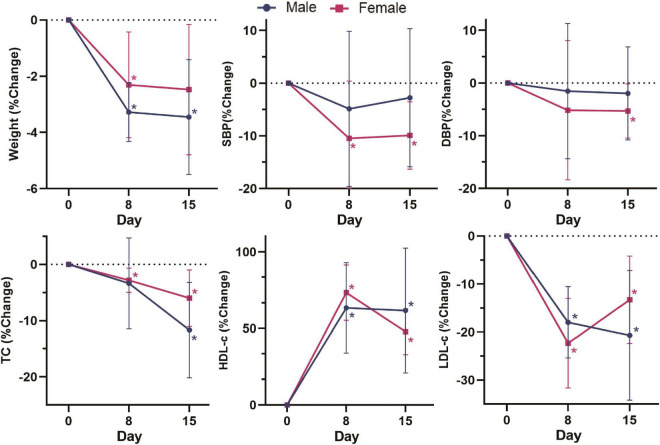
Change (%) in weight, SBP, DBP, TC, HDL-c, and LDL-c in different sex groups on day 0, day 8 and day 15. Change (%) = (Indicators after intervention – Indicators before intervention) × 100%/Indicators before intervention. Data were shown by the percentage of reduction compared with baseline for the two groups. *: Significant difference compared with baseline. *: *p* < 0.05.

In addition, there were sex differences in these changes (Figure 2). Compared with baseline, the body weight and TC of male volunteers decreased by 3.45% (*p* = 0.028) and 11.67% (*p* = 0.028), respectively, after dietary intervention, which was greater than the decreases in female volunteers of 2.47% (*p* = 0.063) and 6.00% (*p* = 0.028). Male volunteers had more stable LDL-c and HDL-c levels than female volunteers, with a 20.68% (*p* = 0.028) decrease in LDL-c, 61.61% (*p* = 0.018) and an increase in HDL-c after dietary intervention, which was more significant than the improvement in female volunteers by 13.27% (*p* = 0.018) and 47.82% (*p* = 0.018). In the second week, the LDL-c and HDL-c levels in female volunteers were markedly reduced in general. The blood pressure level of male volunteers fluctuated less than that of female volunteers. By the end of the intervention, the SBP and DBP levels of female volunteers decreased by 9.91% (*p* = 0.028) and 5.28% (*p* = 0.043), respectively, which was greater than the levels of male volunteers by 2.75% (*p* = 0.498) and 1.96% (*p* = 0.499). In summary, these results indicated the positive effect of our designed dietary pattern on reducing CVD risks.

The above results indicate that the Guangxi longevity dietary pattern constructed in this study could significantly improve the volunteers’ weight, BMI, blood pressure and lipid levels in the short term (2 weeks), with a significant effect on reducing the risk of CVD in volunteers.

### Effects on Fecal Untargeted Metabolomics After 2 Weeks Diet Intervention

**FIGURE 3:**
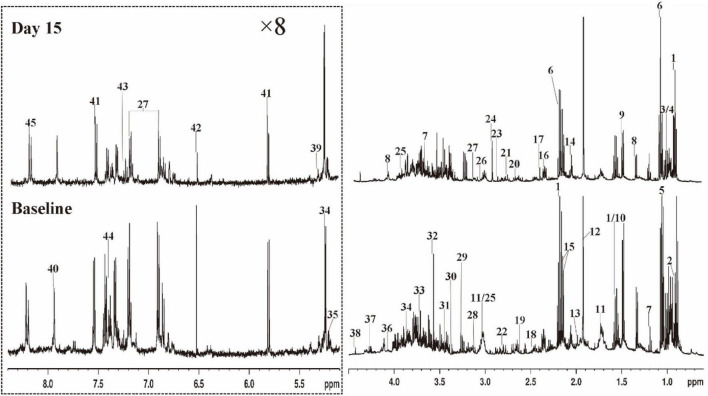
Typical 500 MHz ^1^H NMR spectra of fecal extracts for a volunteer with Baseline and Day 15. The spectra in the region (δ 5.0–8.4) were vertically expanded by 8 times compared with the region (δ 0.5–4.6). 1 butyrate; 2 a-ketoisocaproate; 3 isoleucine; 4 leucine; 5 valine; 6 propionate; 7 ethanol; 8 lactate; 9 alanine; 10 citrulline; 11 lysine; 12 acetate; 13 proline; 14 N-acetylglutamic acid; 15 methionine; 16 glutamate; 17 succinate; 18 glutamine; 19 methylamine; 20 aspartate; 21 dimethylamine; 22 asparagine; 23 trimethylamine; 24 N.N-dimethylglycine; 25 creatine; 26 creatinine; 27 tyrosine; 28 histidine; 29 β-glucose; 30 methanol; 31 taurine; 32 glycine; 33 glycerol; 34 α-glucose; 35 α-xylose; 36 choline; 37 threonine; 38 1.3-dihydroxyacetone; 39 α-arabinose; 40 xanthine; 41 uracil; 42 fumarate; 43 3-hydroxyphenylacetic acid; 44 phenylalanine; 45 hypoxanthine.

**FIGURE 4:**
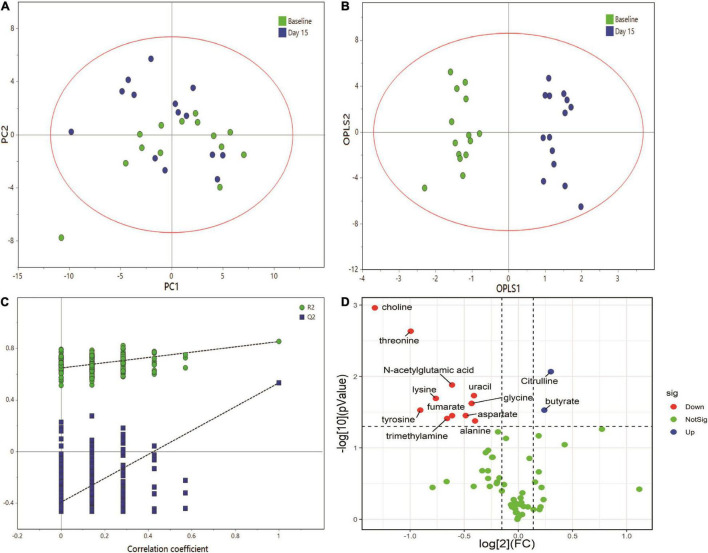
Multivariate data analysis of fecal ^1^H NMR data. **(A)** Score plot of PCA model, **(B)** score plot of OPLS model, **(C)** permutation test (permutation no.: 200), **(D)** The volcano plot of metabolites identified by ^1^H NMR.

Figure 3 shows representative 500 MHz ^1^H-NMR spectrograms of fecal samples before and after dietary intervention. According to the chemical shift and signal diversity of each metabolite, 45 different metabolites were identified and quantified, in combination with the previously reported literature and the human metabolome database (HMDB; http://www.hmdb.ca/) (26–28). To obtain an overview of fecal metabolites before and after dietary intervention, PCA was performed on the results. Figure 4A shows the PC1 and PC2 scores for all samples. The first two principal components cumulatively explained 44.1% of the total variances. To achieve maximum separation between groups in fecal metabolites before and after dietary intervention, OPLS-DA was used to visualize metabolite differences (Figure 4B). The results showed that the model had a good performance for the separation of fecal metabolites before and after dietary intervention in volunteers, R^2^X = 0.759, R^2^Y = 0.945, Q^2^ = 0.592, which indicated that the fecal metabolites before and after dietary intervention in volunteers could be well-identified and distinguished by the model. The Permutation Test (200 times) was used to further evaluate the robustness of the model, R^2^ = 0.641, Q^2^ = −0.383, suggesting that the model was well-fitted and highly predictable. As the R^2^ and Q^2^ values in the permutation test were lower than the corresponding original values, the regression line of Q^2^ had a negative intercept (Figure 4C).

The volcano plot was employed to identify–potentially important metabolic markers according to fold change (FC ≥ 1.1 or ≤0.9) and Paired Samples Wilcoxon test *p* < 0.05 (Figure 4D). Then further combined with the VIP value (VIP > 1) in OPLS-DA score plots, significantly different metabolite profiles were screened and selected as potential biomarkers to determine the essential changes in fecal metabolites before and after dietary intervention (Table 2). Nine metabolites showed significant changes compared with pre-intervention values. Among them, two metabolites increased (butyrate and citrulline); while seven metabolites decreased (threonine, choline, lysine, glycine, aspartate, alanine, and N-acetylglutamic acid).

**TABLE 2:** Statistical analysis results of the main metabolite in feces.

Metabolite (peak position)	VIP	Relative intensity		FC	*p* Value
		Baseline	Day 15		
Threonine (4.24 ppm)	1.57	0.60 ± 0.34	0.30 ± 0.23↓	0.50	0.004
Choline (4.06 ppm)	1.14	0.24 ± 0.16	0.09 ± 0.06↓	0.40	0.001
Glycine (3.57 ppm)	1.57	1.15 ± 0.34	0.85 ± 0.24↓	0.74	0.022
Lysine (3.03 ppm)	2.32	2.90 ± 3.10	1.71 ± 1.06↓	0.59	0.022
Aspartate (2.68 ppm)	1.04	0.56 ± 0.19	0.40 ± 0.21↓	0.71	0.035
N-Acetylglutamic acid (2.06 ppm)	1.60	0.93 ± 0.38	0.61 ± 0.29↓	0.65	0.012
Citrulline (1.56 ppm)	2.46	4.22 ± 1.27	5.20 ± 1.41↑	1.23	0.012
Alanine (1.48 ppm)	2.30	2.87 ± 0.80	2.18 ± 0.70↓	0.76	0.041
Tyrosine (3.07 ppm)	0.81	0.27 ± 0.27	0.14 ± 0.10↓	0.53	0.030
Trimethylamine (2.89 ppm)	0.56	0.14 ± 0.08	0.09 ± 0.05↓	0.63	0.039
Uracil (5.81 ppm)	0.84	0.38 ± 0.12	0.29 ± 0.09↓	0.75	0.019
Fumarate (6.52 ppm)	0.48	0.12 ± 0.08	0.08 ± 0.03↓	0.65	0.035
Butyrate (0.90 ppm)	2.64	7.08 ± 1.93	8.36 ± 2.16↑	1.18	0.030

*“↑” indicates that relative abundance of metabolites increased after the dietary intervention.*

*“↓” indicates that relative abundance of metabolites decreased after the dietary intervention.*

### Correlations Between Feces Metabolites and Clinical Parameters

**FIGURE 5:**
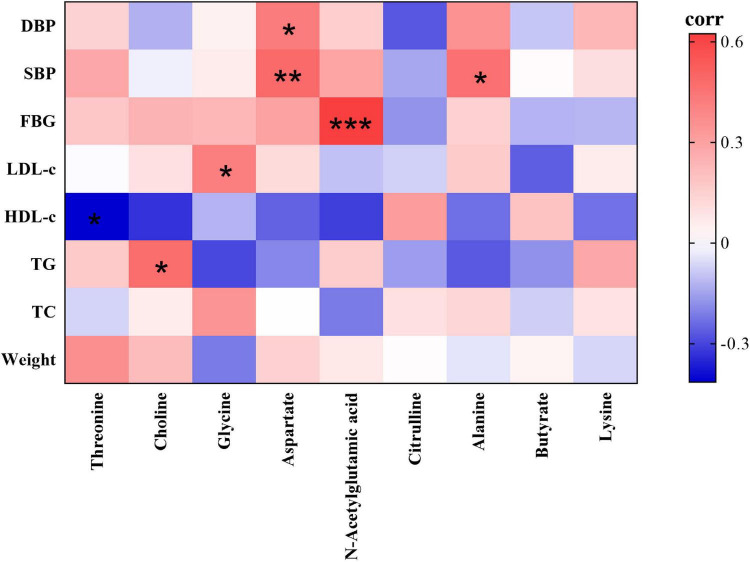
Heatmap of correlations between the nine significantly different fecal metabolites identified and clinical parameters (red indicates a positive correlation, green indicates a negative correlation). Significantly correlated at *p < 0.05, **p < 0.01, ***p < 0.001 (two-sided test).

As the volunteers’ body weight, blood pressure and blood lipid significantly improved after the dietary intervention, and volunteers’ fecal metabolites also significantly changed, it is necessary to further study the correlation between the volunteers’ fecal metabolites and clinical parameters using Pearson correlation analysis. As shown in Figure 5, FBG, blood pressure and serum TG, LDL-c levels were significantly and positively correlated with fecal metabolites in volunteers, of which SBP levels were correlated with alanine and aspartate, DBP levels were correlated with aspartate, FBG levels were correlated with N-acetylglutamic acid, TG levels were correlated with choline, and LDL-c levels were correlated with glycine. Moreover, HDL-c levels were significantly inversely correlated with threonine.

### Prediction of Metabolic Interactions by Search Tool for Interactions of Chemicals

**FIGURE 6:**
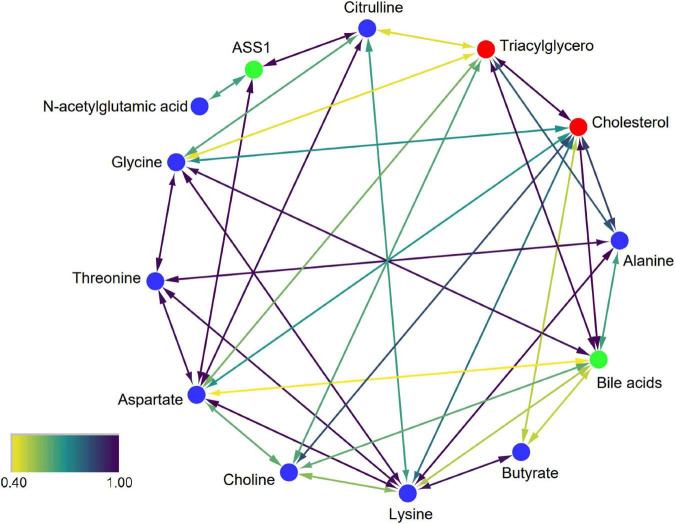
Interaction network of biomarkers. Blue, red, and green nodes represent biomarkers, CVD-related targets, and intermediate chemicals, respectively. The color scale represents the strength of confidence. A higher value represents a stronger data-support interaction.

To explore the exact effects of dietary patterns on the efficacy of reducing CVD in humans, using lipid-related indexes (triacylglycerol and cholesterol) as targets, the relationship between the nine significantly changed biomarkers and targets were explored. The algorithm used by STITCH contains metabolic pathways, molecular structures, etc., which can identify data and their interactions from multiple perspectives, and outperforms other traditional correlation-based network analyses (25). Therefore, metabolic networks based on these biomarkers and biochemical targets were constructed by STITCH. The results showed that all biomarkers can directly or indirectly interact with triacylglycerol and cholesterol (Figure 6). Furthermore, glycine, choline, alanine, lysine, and aspartate showed strong direct interactions with cholesterol (combined score: 0.708; 0.847; 0.872; 0.766; 0.698). Butyrate has a weaker direct interaction with cholesterol (combined score: 0.490). Additionally, choline, alanine, and aspartate also showed strong interactions with triacylglycerol (combined score: 0.637; 0.819; 0.586). Glycine and citrulline had weaker direct interactions with triacylglycerol (combined score: 0.428;0.435). N-acetylglutamic acid and threonine do not directly act with triacylglycerol or cholesterol. However, N-acetylglutamic acid can modulate triacylglycerol and cholesterol via ASS1 to generate citrulline and aspartate. Threonine can modulate triacylglycerol and cholesterol through alanine, aspartate, and glycine. In addition, choline, butyrate, glycine, aspartate, lysine, and alanine can also strongly interact with triacylglycerol and cholesterol through bile acids. In summary, the predicted metabolic interaction among the nine identified biomarkers and lipid targets provided a potential explanation for the changes in lipid metabolism/transport caused by dietary interventions, thus improving the clinical indicators related to CVD in volunteers.

### Metabolic Pathway Analysis

**FIGURE 7:**
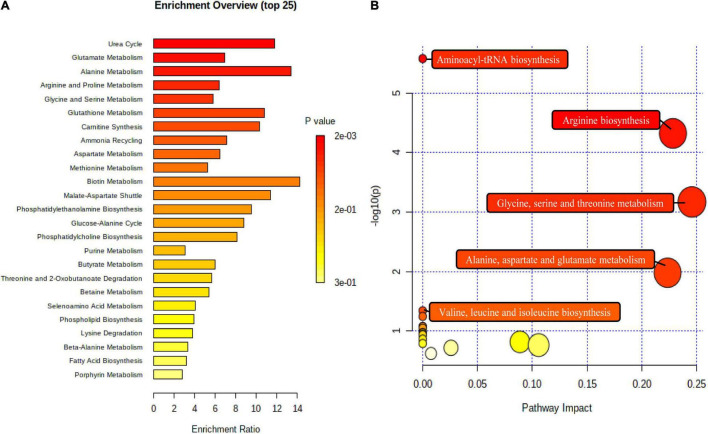
The metabolome view above shows all matched pathways according to *p* values from pathway enrichment analysis **(A)** and pathway impact values from pathway topology analysis **(B)**. The bubble size is proportional to the impact of each pathway and the bubble color denotes the significance, from highest in red to lowest in white.

To understand whether the fecal metabolite changes of volunteers reflected synergistic changes in their metabolic pathways, nine biomarkers were used for metabolome enrichment analysis and pathway analysis using MetaboAnalyst 5.0 (Figures 7A,B). The pathway analysis module of MetaboAnalyst 5.0 contains new algorithms and concepts with the high-quality KEGG and well-established methods. Accordingly, the pathway library of Homo sapiens and Fisher’s exact test was applied for pathway enrichment analysis, and relative-betweenness centrality was performed for pathway topology analysis based on reported protocols (29). Metabolites that have been significantly altered are mapped onto likely relevant pathways, and were used to explain the metabolism. The influenced metabolic pathways were set as *p* value less than 0.05. The results showed that there were five potential metabolic pathways with significant changes that may be critical in reducing the risk of CVD (*p* < 0.05) (30). These were: (1) arginine biosynthesis; (2) aminoacyl-tRNA biosynthesis; (3) glycine, serine, and threonine metabolism; (4) alanine, aspartate, and glutamate metabolism; (5) valine, leucine, and isoleucine biosynthesis. These results indicated that the effects of this dietary pattern on the body’s health status were associated with different metabolic pathways, and these metabolic pathways may play an important role in reducing the risk of CVD in humans.

## Discussion

Studies have shown that a healthy dietary pattern can reduce the risk of CVD and plays an important role in health care (8, 9). However, the application and promotion of these healthy dietary patterns were limited by the differences in food types and eating habits in different areas. Therefore, it is necessary to combine the characteristics of local food materials and dietary habits to study healthy dietary patterns with high local acceptance in different regions. This small pilot study showed that 2 weeks intervention using the Guangxi longevity dietary pattern was beneficial for reducing CVD risk factors in humans under high compliance, which was characterized by significantly reduced body weight, TC, LDL-c, and blood pressure levels, while it increased volunteers’ HDL-c levels. Untargeted fecal metabolomics analysis demonstrated that nine metabolites showed significant differences, including amino acid metabolism, lipid metabolism and urea cycle. Among them, two metabolites increased (butyrate and citrulline), seven metabolites decreased (threonine, choline, glycine, aspartate, alanine, N-acetylglutamic acid, and lysine). We also found that five pathways are closely related to these beneficial effects, involving the biosynthesis of arginine, aminoacyl-tRNA, valine, leucine and isoleucine, as well as the metabolism of alanine, aspartate and glutamate, glycine, serine and threonine.

### Anthropometry and Blood Biochemical Metabolism

As important factors affecting individual lifespan, body weight and BMI are significantly associated with CVD and mortality risk (31). This relationship was particularly obvious among Asians. Studies have shown that a one-unit increase in BMI would cause 14% of the increase in the risk of coronary heart disease (32). In this study, volunteers’ averaged baseline BMI > 25 kg/m^2^, therefore, it is very important to apply personalized weight loss strategies for overweight volunteers to regulate their health status. After 2 weeks of the high-compliance Guangxi longevity dietary intervention, volunteers’ body weight was significantly reduced (2.96%, *p* = 0.004), and BMI decreased by 0.79 units (25.42 ± 3.75 vs 24.63 ± 3.35 kg/m^2^, *p* = 0.004). This may be related to our dietary pattern, with rice and coarse cereals as the staple food, which was rich in fruits and vegetables and has a high dietary fiber content. Studies have shown that a higher dietary fiber intake helps to reduce food energy density, increase satiety, and buffer fluctuations in insulin levels, thereby effectively regulating appetite (33). Therefore, the Guangxi longevity dietary can reduce the body weight and BMI of volunteers and better protect the body’s health state.

This dietary pattern also significantly improved blood lipid levels in the volunteers. After the dietary intervention, TC and LDL-c levels were reduced by 8.84% (*p* = 0.002) and 16.98% (*p* = 0.001), and HDL-c level increased by 54.71% (*p* = 0.001). The blood lipid levels can reflect the lipid metabolism status in the body. It has been widely used in the prevention and treatment of CVD, and thus has become an important indicator of human health status (34, 35). The potential mechanism regulating these may be the diet containing nuts rich in essential fatty acids such as ω-3 fatty acids, arachidonic acid, and a high intake of unsaturated fatty acids. Studies have shown that the appropriate intake of polyunsaturated fatty acids (PUFA) can effectively reduce blood TC and LDL-c levels, with positive effects on reducing CVD risk and improving body metabolism (36, 37). Meanwhile, high levels of soluble dietary fiber intake are also a key factor affecting blood cholesterol levels. The dietary fiber content in this study is high, soluble dietary fiber intake could effectively accelerate gut motility, increase the fecal excretion of bile acids, and reduce bile acid reabsorption in the small intestine. Organism bile acid loss would lead to LDL-c uptake by the liver from the blood, which would reduce serum TC and LDL-C concentrations (38). Therefore, Guangxi longevity dietary pattern can effectively improve blood lipids and reduce the body’s risk of CVD.

Hypertension is one of the main causes of CVD and death. Healthy dietary patterns have been shown to be effective in controlling blood pressure, which then reduces CVD risk (39). The Guangxi longevity dietary pattern constructed by our team showed a significant decrease in SBP (6.33%, *p* = 0.041) and DBP (3.62%, *p* = 0.044) levels of volunteers after 2 weeks of high compliance dietary intervention. Dietary patterns rich in potassium, magnesium and dietary fiber, while limiting total fat intake, have been shown to have a good blood pressure reduction effect (40). This is consistent with our study showing that Guangxi longevity dietary pattern was low in fat, and high in potassium, magnesium and dietary fiber due to its high levels of whole grains, fruits and vegetables. These results also indicate the potential protective effect of this dietary pattern on the human cardiovascular system.

Although volunteers’ FBG levels showed a decreased trend after the dietary intervention, there was no statistical difference. This might be due to the limited number of volunteers and their different FBG levels, so short-term dietary intervention had a limited effect on FBG. In addition, according to the existing dietary intervention studies, the hypoglycemic effect of diet needs to be further explored (41).

### Untargeted Feces Metabolomics and Metabolites

In this study, nine metabolites with significant differences were identified by ^1^H-NMR (Table 2), mainly involving amino acid metabolism, lipid metabolism and urea cycle. Moreover, serum TG, LDL-c, HDL-c, FBG, and blood pressure levels were significantly correlated with fecal metabolites. The STITCH network showed that all metabolic markers could directly or indirectly interact with triacylglycerol and cholesterol, suggesting that the effect of the dietary pattern on improving the risk of CVD in volunteers was closely related to the changes in metabolic markers. Pathway and enrichment analysis showed that dietary intervention mainly affected five pathways, including the biosynthesis of arginine, aminoacyl-tRNA, valine, leucine, and isoleucine, as well as the metabolism of alanine, aspartate and glutamate, glycine, serine, and threonine.

#### Amino Acid Metabolism

After the dietary intervention, some amino acid levels in the fecal metabolites of volunteers were markedly decreased (threonine, glycine, lysine, aspartate, and alanine). Many studies have demonstrated that fecal amino acids are effective in predicting organism health status, with good diagnostic potential in diseases such as diabetes (42). Studies have shown that subjects at high risk of diabetes have higher levels of fecal amino acid, and that the impaired metabolism of the organism caused by obesity also leads to an increase in fecal amino acid levels. For example, higher levels of alanine have been found in the feces of overweight and obese subjects (43). Studies in patients with diabetes and metabolic syndrome have found that the concentration of fecal alanine increased with the development of the disease (44). And alanine was positively associated with dyslipidemia (45). It was also verified in the animal model that diabetic mice exhibited higher levels of aspartate and lysine in feces than healthy mice (46). All volunteers in this study were at risk for CVD, so we conclude that a reduction in fecal amino acid levels is a marker of recovery from metabolic adjustment and has a positive effect on improving blood lipids and blood glucose.

Meanwhile, the nutrient malabsorption caused by intestinal epithelial inflammation and injury leads to excessive amino acid levels in feces (47). As an essential amino acid, threonine is an important component of intestinal mucin proteins, and can effectively help the intestine to form mucus gels, thereby protecting the intestine from pathogens. It can also improve the immune system by participating in inflammation and immune gene expression (27). Significantly higher levels of threonine were found in the fecal metabolites of patients with irritable bowel syndrome (IBD) and ankylosing spondylitis (AS) (27, 48). Meanwhile, increased levels of lysine, alanine, and glycine have also been shown to correlate with malabsorption due to intestinal inflammation (49, 50). Therefore, the decreased fecal levels of alanine, threonine, aspartate, lysine and glycine could reflect the improved inflammatory status *in vivo*. In addition, threonine and lysine in the intestine can synthesize butyrate, thus improving the lipid and blood pressure levels (51).

#### Lipid Metabolism

Butyrate and choline, as important components of lipid metabolism, also significantly changed after intervention. As the most common short-chain fatty acids (SCFAs), butyrate is mainly produced by the fermentation of indigestible carbohydrates by gut microorganisms (52). Studies have shown that butyrate can effectively improve blood pressure status, and play a positive role in maintaining the integrity of intestinal epithelial cells and reducing apoptosis (53, 54). And butyrate can increase insulin sensitivity by stimulating intestinal epithelial cells to secrete glucagon-like peptide 1 (55), and improve blood lipid levels by down-regulating the expression of nine key genes of cholesterol biosynthesis (56). It has been demonstrated that short-term dietary interventions can rapidly change the composition and activity of gut microbiota, thereby affecting the production of SCFAs (57). After the dietary intervention, butyrate levels in the volunteers’ feces significantly increased. The potential reason for this is that the high levels of grains, fruits and vegetables in the diet effectively modify the composition and numbers of the bacterial communities (58). Therefore, Guangxi longevity dietary pattern is able to play a positive role in reducing the risk of CVD by increasing butyrate content.

Choline, as part of the lipid metabolism, was significantly reduced after this experiment. In the large intestine, choline can be converted to trimethylamine (TMA) by gut microbiota, and subsequently delivered to the liver through the portal circulation, then metabolized to trimethylamine-N-oxide (TMAO) in the liver and circulated throughout the body (59). TMAO is a predictor of CVD risk such as coronary heart disease, atherosclerosis, etc. (60). Studies also demonstrated a significant dose-dependent association between TMAO concentration and the risk of hypertension, which is also associated with cardiovascular metabolic risk indicators such as HDL-c in both healthy individuals and patients with CVD (61). Therefore, we believe that a significant reduction in choline levels plays an important role in reducing the risk of CVD.

#### Urea Cycle

Although the urea cycle occurs in the liver, studies have shown that citrulline in the body is almost exclusively produced by the gut (62). As a precursor substance for arginine, the citrulline produced in the intestine can be released into the blood stream, becoming a major source of endogenous arginine (63). In addition, arginine metabolism in the intestine can also generate citrulline, which is released into the portal vein and participates in urea cycle processes (64). N-acetylglutamic acid as an essential cofactor of carbamoyl phosphate, studies have proved that the synthesis of citrulline and urea is related to it (65). Therefore, we believe that the changes of citrulline and N-acetylglutamic acid in volunteers’ feces are associated with urea cycle.

As essential components of the arginine biosynthetic pathway, Studies have proven that citrulline may effectively protect diabetic patients from renal damage by promoting nitric oxide (NO) production. Meanwhile, NO produced from citrulline increases the adenosine monophosphate-activated protein kinase alpha (AMPKα) of phosphorylation and activation, then specifically inhibiting the expression of sterol regulatory element-binding protein 1 (SREBP-1). SREBP-1 makes a master regulator of lipogenesis, which can induce cholesterol and fatty acids biosynthesis. Therefore, citrulline mediated inhibition of SREBP1 expression would decrease lipogenesis and accumulation (66). In addition, it has anti-inflammatory properties, and can exert beneficial effects on a variety of metabolic problems caused by aging (67). Therefore, the significant increase in citrulline in this study has a positive effect on reducing blood lipids and improving body health. N-acetylglutamic acid plays an important role in the urea cycle. Although N-acetylglutamic acid has not been reported to have the ability to improve CVD risk, STITCH predicted a close association between N-acetylglutamic acid and citrulline. Thus, the mechanism behind this was likely related.

In summary, nine of the metabolites discussed above played an important role in reducing the risk of CVD in the human body. Our study had high compliance and low dropout (*n* = 1) throughout the experiment, which shows that the designed dietary pattern was well-tolerated. This study has some limitations. The intervention time is short, which may have weakened its ability to detect changes in the studied parameters. Therefore, it is necessary to extend the intervention time in subsequent experiments to further evaluate the improvement effect of this dietary pattern on CVD risk factors, and establish an effective pathway for CVD disease prevention through diet.

## Conclusion

Human health indicators and ^1^H NMR-based metabolomics analysis revealed that Guangxi longevity dietary pattern has the beneficial property of reducing CVD risk factors at a short-term intervention with high compliance. This study better verified and highlighted the beneficial effect of Guangxi longevity dietary pattern on human health, which provides a new strategy for ensuring physical health and promoting optimal cardiometabolic through food instead of drugs. This has a promising development prospect. To support these results, further in-depth investigations are necessary, and targeted metabolomics analysis could also be performed.

## Data Availability Statement

The raw data supporting the conclusions of this article will be made available by the authors, without undue reservation.

## Ethics Statement

The studies involving human participants were reviewed and approved by The Ethics Committee of Guangxi University (Approval No.GXU-2020-136). The patients/participants provided their written informed consent to participate in this study.

## Author Contributions

KH and QL: conceptualization. JM and JD: software. DH: formal analysis. WZu and XY: investigation. WZe, YS, and FS: data curation. KH: methodology and Writing—original draft. DH and QL: writing—review and editing. All authors had reviewed the manuscript and approved the final manuscript.

## Conflict of Interest

The authors declare that the research was conducted in the absence of any commercial or financial relationships that could be construed as a potential conflict of interest.

## Publisher’s Note

All claims expressed in this article are solely those of the authors and do not necessarily represent those of their affiliated organizations, or those of the publisher, the editors and the reviewers. Any product that may be evaluated in this article, or claim that may be made by its manufacturer, is not guaranteed or endorsed by the publisher.
