# Supplementary material for: A Clinical Trial of the Effects of a Dietary Pattern on Health Metrics and Fecal Metabolites in Volunteers With Risk of Cardiovascular Disease
Source: Front Nutr. 2022 May 10;9:853365. doi: 10.3389/fnut.2022.853365 (PMC9128613; doi:10.3389/fnut.2022.853365)
Supplement: Supplementary file 1 [file Table_1.DOCX]

Supplementary Material

**Table S1.** Food grouping used in the dietary pattern

| Food group | Food items |
| --- | --- |
| White rice | White rice |
| Cereal | Black rice, oat, corn grits, etc. |
| Noodle | Noodles |
| Flour | Wheat flour, cornstarch |
| Tuber | Potatoes, taros, yams, etc. |
| Legumes | Beans, tofu, dried bean curd, etc. |
| Nuts and Seeds | Peanuts, almonds, walnut kernels, pumpkin seeds |
| Vegetables | Carrots, tomatoes, cabbages, etc. |
| Mushroom | Mushroom, fungus |
| Fruits | Guava, bananas, apples, etc. |
| Red meats | Pork |
| Poultry | Chicken, duck |
| Eggs | Eggs |
| Fish | Perch, dried shrimp |
| Seaweeds | Laver |
| Plant oils | Sesame oil, peanut oil |
| Fats | Animal fats |
| Seasonings | Soy sauce, vinegar, sugar, etc. |

**Table S2.** Example of a one-day menu during the intervention^1^

|  | **Food** |
| --- | --- |
| Breakfast | 280g Red bean oatmeal porridge |
|  | 180g Sweet potato |
|  | 55g Boiled egg |
| Lunch | 155g Rice |
|  | 130g Broccoli |
|  | 32.5g Chicken breast |
|  | 150g Bitter dish |
|  | 23g Dried bean curd |
|  | 40g Black fungus |
|  | 63g Carrot |
|  | 40g Sweet pepper |
|  | 250g Peanut Lotus Root Corn Soup |
| Dinner | 100g Rice |
|  | 130g Pakchoi |
|  | 31g Mushroom |
|  | 130g Cabbage |
|  | 49g Chicken  150g Oyster mushroom |
|  | 25g Green pepper |
|  | 220g Corn porridge |
| Snacks | 200g Banana |
|  | 200g Orange |
|  | 16g Walnut kernel |

^1^Friday menu of the 1678 kcal/d energy level
